# Data Mining and Polar Coordinates in the Analysis by Gender of Finishing Behaviors in Professional Basketball Pick and Roll

**DOI:** 10.3389/fspor.2021.742609

**Published:** 2021-12-08

**Authors:** Juan Pablo Morillo-Baro, Belén Troyano-Gallegos, José Luis Pastrana-Brincones, Juan Antonio Vázquez-Diz, Rafael E. Reigal, Yarisel Quiñones-Rodríguez, Antonio Hernández-Mendo, Coral Falcó, Verónica Morales-Sánchez

**Affiliations:** ^1^Department of Social Psychology, Social Work, Anthropology and East Asian Studies, University of Málaga, Málaga, Spain; ^2^Department of Languages and Computer Science, University of Málaga, Málaga, Spain; ^3^Department of Sports Dididactics, University of Pinar del Río Hermanos Saín Montes de Oca, Pinar del Río, Cuba; ^4^Department of Sport, Food and Natural Sciences, Western Norway University of Applied Sciences, Bergen, Norway

**Keywords:** mixed method, systematic observation, polar coordinates, data mining, basketball

## Abstract

The open nature of basketball gives it a large uncertainty that makes hard the tactical analysis of the situations that happen in the game. Specifically, screens are one of the offensive tactical elements most used in basketball and one example of a tactical situation that needs the highest preparation level to get a good performance in the competition. The aim of this study is to differentiate these player behaviors by gender using data mining and polar coordinates analysis. Therefore, one *ad hoc* observational tool made by 17 criteria and 97 exhaustive and mutually exclusive (E/ME) categories has been designed and validated using the data quality analysis (correlation coefficients and concordance index 0.98) and generalizability analysis (G coefficients 0.94) to perform such a study. The observational design is nomothetic, punctual, and multidimensional. A total of 176 ball screens situations have been analyzed for the men's category and 118 for women's category, corresponding to three different teams of each gender playing in the highest competition level in Spain during the 2018/2019 season using Hoisan software tool. The analysis of the relationships among behaviors has been performed using Polar Coordinates analysis as well as data mining analysis: clustering and decision tree classifier. Results show significant relationships that allow us to tactically interpret the pick and roll situations in men's and women's professional basketball players in Spain, allowing us to develop more intervention programs which will optimize training and improve players performance.

## Introduction

Cooperative-opposition collective sports, such as basketball, are characterized by a high motor interaction and a high degree of uncertainty in those interactions (Castellano and Hernández Mendo, 2000; Castellano et al., 2009; Conejero et al., 2016) because during a game there are interactions with both teammates and opponents. Therefore, although it is a sport that has been studied from many fields of knowledge such as biomechanics (Morales Toapanta et al., 2018), psychology (Rodríguez López and Sáez Rodríguez, 2009), or physiology (Calleja et al., 2008), it is important that it also be analyzed from a perspective where motor interaction is taken into account (Hernández-Mendo et al., 2000) because the protagonists are the players and their technical–tactical reading.

In recent decades, basketball has experienced a progressive increase in physical, technical, and tactical demands in competition (Sarmento et al., 2017). Together with the study of the game situations carried out by the technical staff, has made harder to obtain offensive advantages to score points and perform at the highest level. So it is tried to take advantage from each phase of the game to improve the performance (Morillo-Baro et al., 2020). High level of intensity requires an optimal physical preparation and an extensive technical–tactical background for the players that allows them to adapt to the continuously changing environments they face (Morillo-Baro et al., 2020).

Pick and roll is a collective offensive resource frequently used in the offensive phase throughout the game, being present in ~30–45% of positional attacks (Nunes et al., 2016; Romarís Durán, 2016). Numerous studies have investigated the importance of this tactical procedure in high-level basketball (Koutsouridis et al., 2018; Nunes et al., 2021; Sekulić, 2021). It is so important that it is the most used action to end attacks in Liga ACB, and the second most used, after the individual play, in Liga Femenina (Romarís Durán et al., 2013). It is defined as the collective action of the two-on-two (2 × 2) game in which the attacker without the ball performs a screen (hinders an opponent by placing himself in his path) helping his/her mate who is having the ball (Cárdenas, 2010; Muñoz Arroyave et al., 2015; Nunes et al., 2016). Therefore, studying it combined with other tactical behaviors is a matter of great interest for coaches who aim to optimize the game performance of the team (Nunes et al., 2016; Morillo-Baro et al., 2020). It is also important to study the behavior in training situations, during formative stages with young players, in line with the studies developed by Mateus et al. (2019, 2020), modifying the dimensions of the game space or in simulated game situations.

Mixed methods and observational methodology has got high importance in recent decades in the sport context (Anguera and Hernández-Mendo, 2015; Anguera et al., 2021). Its applicability in the natural context of high-performance sport creates an optimal relationship between science and practical application, focusing on the spontaneous and habitual behavior of the observed participants (Anguera, 1990; Anguera and Hernández-Mendo, 2013; Sánchez-Algarra and Anguera, 2013). Added to the possibility of developing *ad hoc* observation instruments (Sarmento et al., 2010) adapted to the reality of a given context, it allows a detailed analysis of the behaviors involved in the tactical development of the game.

This combination of quantitative and qualitative data represents the essence of mixed methods, transforming data from qualitative to quantitative and vice versa, and interpreting the results after analysis (Cresswell and Plano Clark, 2011). An example of this is the observational methodology itself (Anguera and Hernández-Mendo, 2016) together with its distinctive analyses such as polar coordinates analysis (Vázquez-Diz et al., 2019; Morales-Sánchez et al., 2020) and also data mining (Pastrana et al., 2019). These techniques have shown to be very useful in sport psychology to explore several variables usually studied in this field of knowledge, getting useful information on how scores are distributed and groups are classified (Pastrana et al., 2019; Vázquez-Diz et al., 2019; Reigal et al., 2020), and studying the relationships established between the behaviors that occur (Nunes et al., 2016; Morillo-Baro et al., 2020).

Data mining is considered a set of techniques and technologies that allows extracting useful information from a large data set, such as patterns and trends. It attempts to find patterns of behavior in large data sets to explain them (Pastrana et al., 2019). Clustering is one of the most used techniques in data mining. Basically, it is the task of dividing the population into a number of groups such that elements in the same group are more similar but different from those in the other groups. In other words, the aim is to segregate groups with similar traits and assign them into clusters. The k-means clustering algorithm (Likas et al., 2003) is one of the most used algorithms for clustering, to cluster observations into groups of related observations without any prior knowledge of those relationships. Thus, it becomes a very useful tool because it allows us to find or identify unknown groups that are often not otherwise identified (Zaki and Meira, 2014; Witten et al., 2016).

This clustering technique is also used as a prestep for several algorithms, such as “classification” or “attribute selection,” which would perform better and faster on a smaller selected set of attributes (Dutt et al., 2015; Thomas et al., 2018). The J48 algorithm (Bhargava et al., 2013) is used to classify different data to get accurate results of the classification. The J48 algorithm is one of the best machine learning algorithms to examine the data categorically and continuously. The J48 decision tree algorithm is a classification tool (Kaur et al., 2015) that creates an acyclic graph structure (a tree), where attributes are represented at internal nodes and arcs, representing how the values are divided. Each leaf node will be a value of the target class. Decision trees are often built from a training set and then used as a model of the problem to predict future behavior.

On the other hand, the polar coordinate technique uses a sequential prospective and retrospective lag analysis of the recorded behaviors (Sackett, 1980; Anguera et al., 1997). It allows a drastic reduction of the analyzed data and a graphical representation of the established relationships between focal and conditioned categories through a vector system (Hernández-Mendo and Anguera, 1999). The contrast statistic of this analysis is the *Z*sum (*Z*sum = Σ*z*/√*n*, where *n* is the number of lags) (Cochran, 1954). The distribution of this parameter *Z*sum has a *x* = 0 and an Sx = 1. The relationships between behaviors and their vector representation are obtained from these values. A value is considered statistically significant when the vector module is equal or greater than 1.96. This value is estimated through the square root of the sum of the square of the *Z*sum of the *X* (prospective) and the square of the *Z*sum of the *Y* (retrospective):


$$\begin{array}{l} Module=\sqrt{ZsumP^{2}+ZsumR^{2}} \end{array}$$

The angle of the vector (ϕ = Arc sine of *Y/*Radius) will determine the excitatory or inhibitory nature of the relationship (Castellano and Hernández-Mendo, 2003).

Considering the possibilities offered by data mining and polar coordinates analysis, the objective of this work is to extract patterns from the data to explain and predict the behaviors occurring in the execution of the tactical means of ball screen in professional basketball in Spain, differentiating it according to gender.

## Materials and Methods

### Research Design

This study has been carried out using a design located in the fourth quadrant of a nomothetic, punctual, and multidimensional nature, according to the proposal of Anguera et al. (2011). Nomothetic due to the analysis of several teams, punctual because it is the registration of several games but of different teams in the regular phase, and multidimensional because several dimensions have been observed that correspond to the various criteria of the observational tool. Furthermore, since the observer does not interact with the subjects, it is a nonparticipant observational process (Anguera et al., 2000).

### Participants

In this work, three men's teams (FC Barcelona Lassa, Unicaja and Valencia Basket) of the ACB league and three women's teams (Perfumerías Avenida, Girona FC and Lacturale Araski) of basketball of the 2018–2019 season were chosen, and a total of three games of each category in the regular phase were analyzed. Finally, 176 ball screen situations from the ACB League and 117 ball screen situations from the Women's League were observed and coded.

According to the Belmont Report and the standards of competence, since the videotapes that we have analyzed for this study are in the public domain, it has not been necessary to obtain informed consent from the participants. The Belmont Report describes basic ethical principles and guidelines concerning ethical issues in human subject research. According to these guidelines, images of public behavior can be used for research without getting the informed consent of the athletes (American Psychological Association, 2002). We analyzed games belonging to the official professional basketball competition in Spain (ACB League and Women's League) that was broadcast on public television (Movistar+, 0 and TDP), hoping for a capacity audience.

### Instruments

To conduct this work, Hoisan software (Hernández-Mendo et al., 2012, 2014) was used to code and record the behaviors, perform the data quality analysis, and the polar coordinates analysis. The optimization of the graphical representation of the polar coordinates analysis has been performed with the R program (Rodríguez-Medina et al., 2019), and SAGT program (Hernández-Mendo et al., 2016) has been used for the generalizability analysis.

To carry out this work, a first *ad hoc* observational tool, called the Tactical Assessment of Ball Screen in Basketball (VTBDB), was created with the HOISAN software (Hernández-Mendo et al., 2012) with the aim of observing, coding, and analyzing ball screen in basketball. Once this tool was created, several games were observed to improve it. Finally, the tool is composed of 17 criteria and a total of 97 categories, as shown in Table 1.

**Table 1:** Observation instrument for the tactical assessment of ball screen in basketball (VTBDB).

**Criteria**	**Categories**	**Description**	**Criteria**	**Categories**	**Description**
Minute	Q1-1	From min 10 to 5 of the first quarter	Marker	G+10	Winning by more than 10 points
	Q1-2	From min 5 to 0 of the first quarter		G6-10	Winning between 6 and 10 points
	Q2-1	From min 10 to 5 of the second quarter		G1-5	Earned between 1 and 5 points
	Q2-2	From min 5 to 0 of the second quarter		EMP	Tie
	Q3-1	From min 10 to 5 of the third quarter		P1-5	Losing between 1 and 5 points
	Q3-2	From min 5 to 0 of the third quarter		P6-10	Losing between 6 and 10 points
	Q4-1	From min 10 to 5 of the fourth quarter		P+10	Losing by more than 10 points
	Q4-2	From min 5 to 0 of the fourth quarter			
	PR1	Extension 1			
	PR2+	Extension 2 or more			
Possession	24–15	24–15 s of possession remaining	Type of ball screen	SIMP	Simple screen
	14–8	14–8 s of possession remaining		DOBL	Double screen
	−7	Less than 7 seconds of possession remaining			
Player with ball	BLB	Base with ball	Player screening 1	BQ1B	Base screener
	BLE	Escort with ball		BQ1E	Escort screener
	BLA	Eaves with ball		BQ1A	Alero screener
	BLAP	Wing center with ball		BQ1AP	Center Wing screener
	BLP	Pivot with ball		BQ1P	Pivot screener
Player screening 2	BQ2B	Base screener	Zone	Z1	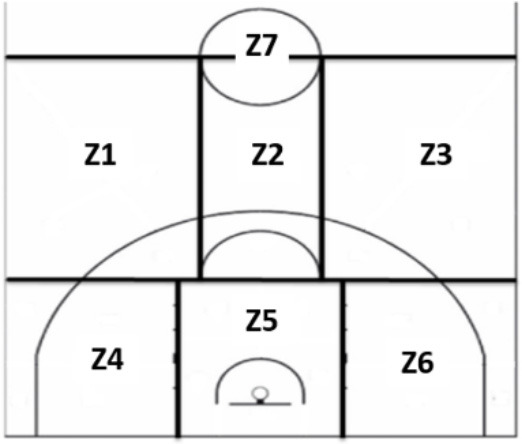
	BQ2E	Escort screener		Z2	
	BQ2A	Alero screener		Z3	
	BQ2AP	Center Wing screener		Z4	
	BQ2P	Pivot screener		Z5 Z6 Z7	
Orientation	OC	Oriented to the center of the field	Ball screen side	BQLD	Dominant side ball screen
	OB	Band oriented		BQLND	Non-dominant side ball screen
	OF	Oriented to the bottom line		BQF	Screener is needed
	WTO	Midfield oriented		BQA	Referee for the game
Exit from the ball screen	SLBQ	Exit on the ball screen side	Defensive system	IND	Individual
	SCBQ	Exit on the opposite side of the ball screen		ZN	Zone
	NBQ	Does not come out of the ball screen		MIX	Mixed
	SBQF	The player with the ball fouls			
	SBQA	The referee for the game			
Defender 1	D1C	Defensor changes defender	Attacker 2	A2PR	Attacker makes pick and Roll
	D1FL	Screener advocate flashes		A2PP	Attacker makes Pick and Pop
	D1N	Defender of the screener does nothing		A2BQ	Attacker ball screens again
	D1F	Defender of the screener is needed		A2F	Attacker needed
	D1A	Referee for the game		A2A	Referee for the game
Defender 2	D2C	Defender of the player with the ball changes	Attacker 1	A1P	Attacker passes the ball
	D2-1	Defender close to the player with the ball		A1T	Attacker makes a shot
	D2-2	Defender behind the screener		A1E	Attacker makes an entry
	D2-3	Defender behind the three players		A1R	Attacker retains the ball
	D2BQ	Defensor remains in the ball screen		A1BE	Static boat
	D2S	Defensor pursues		A1BM	Boat in motion
	D2F	Defensor is needed		A1F	Attacker needed
	D2A	Referee for the game		A1A	Referee for the game
Final	PV	Advantageous pass			
	EV	Advantageous entry			
	TV	Advantageous shot			
	TD	Disadvantageous shot			
	BV	Advantageous boat			
	CONT	The play continues			
	PERD	Loss of possession			
	FD	Foul of the defense			
	FA	Attack foul			
	ARB	Referee for the game			

The observational instrument has passed the data quality tests required by observational methodology. Table 2 shows the results of Kendall's, Pearson's, and Spearman's Tau *b* correlation coefficients, reaching minimum indexes of 0.988, and Cohen's Kappa index shows a minimum value of 0.987.

**Table 2:** Values of correlation coefficients and concordance index.

**Coefficient**	**Intra (Obs. 1 vs. Obs. 1 bis)**	**Concordance inter (Obs. 1 vs. Obs. 2)**
Pearson	0.999	0.999
Spearman	0.991	0.992
Kendall's Tau b	0.988	0.988
Cohen's Kappa	0.987	0.988

The generalizability theory (Cronbach et al., 1972; Cardinet et al., 1976, 1981) allows to control the adequate quality of the data coming from the MO and to establish an accurate estimation of the different sample sizes (Blanco-Villaseñor et al., 2014).

To test intra- and interobserver reliability, a two-facet, category and observer (C/O) design has been used. The results obtained by the SAGT program showed that both intraobserver and interobserver variability was associated with the category facet at 99.39 and 93.32% respectively, being 0 for the observers facet and 0.60 and 6.67% in the interaction of the categories–observer facets. Table 3 shows the values associated with each facet.

**Table 3:** Values associated with each facet in intra- and inter-observer reliability.

	**% [0]**	**%[C]**	**%[O] [C]**
Intraobserver variability	0.000	99.394	0.606
Interobserver variability	0.000	93.321	6.679

A two-facet design, observers and categories (O/C), has been also carried out for the homogeneity analysis of the tool. This design ascertains the degree of differentiation among the different actions of the game using the proposed categories. The results showed that variability is 99.39% associated with the category facet, 0.60% for the observers/categories interaction, and 0.00% for the observer facet. Since the generalization coefficients tend to zero, the homogeneity of the categories can be admitted to be optimal in the sense of differentiators (Blanco-Villaseñor et al., 2014).

The estimation of the minimum number of sessions to be observed to generalize with optimal precision has been performed using a two-facet design, categories and matches (C/P). Finally, an absolute *G* coefficient of 0.949 was obtained when analyzing the three matches. Table 4 shows the obtained values in the G coefficients according to the matches to be observed. It was decided to carry out three observations for each of the categories (men and women).

**Table 4:** Evolution of G coefficients as a function of the number of matches to be observed.

	**2 matches**	**3 matches**
Relative G coefficient	0.932	0.953
Absolute G coefficient	0.925	0.949

### Procedure

Once the tool has been validated and the reliability of the observers has been estimated by means of the data quality and generalizability analyses, the observations of the six matches have been coded and the polar coordinate analyses have been carried out using the HOISAN program (Hernández-Mendo et al., 2012).

First, a sequential analysis of all observations made with the selected focal behavior has been performed, obtaining the *Z* results with a delays range between −5 and 5. Calculations were made to determine the *Z*sum parameters (prospective and retrospective), the quadrant assignment, the module, the angle, and the transformed angle for the rest of the categories (Castellano and Hernández-Mendo, 2003) using these values. The characterization of each quadrant is as follows (Castellano and Hernández-Mendo, 2003):

Quadrant I [+,+]: Criterion behavior is excited with respect to mating behavior in the retrospective and prospective perspective.
Quadrant II [–,+]: The criterion behavior has a relationship with respect to the mating behavior of excitation in retrospective perspective and inhibition in prospective perspective.
Quadrant III [–,–]: The criterion behavior has a relationship with respect to inhibition mating in the retrospective and prospective perspective.
Quadrant IV [+,–]: The criterion behavior has a relationship with the mating behavior of excitation in prospective perspective and inhibition in retrospective perspective.
Two behaviors have been selected as focal (given): Exit on the opposite side of the ball screen (SCBQ) and exit on the side of the ball screen (SLBQ).

Subsequently, the gender-differentiated data matrices have been used for data mining analysis (Weka 3, 2021). This type of analysis has made it possible to establish relationships between attributes or data sets, group similar data, classify attribute relationships, and show information that might be hidden or lost in a large amount of data not mined.

## Results

### Polar Coordinates

The polar coordinates analysis shows the results for each gender (male and female) in two-ball screens, on the one hand, the focal behavior “output on the opposite side of the ball screen” (SCBQ) and on the other hand, the “output on the side of the ball screen” (SLBQ).

Table 5 shows the relationship among the focal behavior SCBQ with the rest of the mating behaviors for all the observations made for each gender.

**Table 5:** Significant relationships and vector representation between SCBQ focal behavior and mating behaviors for each gender.

**C**	**Male**			**Female**		
	**Category**	**Radio**	**Angle**	**Category**	**Radio**	**Angle**
**I**	JCB_BLE	2.18 (^*^)	82.02	MIN_Q2-2	2.4 (^*^)	60.26
	JCB_BLA	3.1 (^*^)	9.66	D2_D2-2	2.08 (^*^)	59.45
	D2_D2-1	2.18 (^*^)	51.13	D2_D2-3	2.05 (^*^)	69.99
**II**	MIN_Q1-1	2.46 (^*^)	92.46	MIN_Q2-1	2.45 (^*^)	140.5
	MARC_G1-5	3.08 (^*^)	94.3	FIN_TD	2.23 (^*^)	111.72
				FIN_PERD	1.99 (^*^)	131.74
**III**	MIN_Q2-1	2.49 (^*^)	260.99	MARC_P6-10	2.02 (^*^)	258.95
	MIN_Q2-2	3.97 (^*^)	229.72	MARC_P+10	2.62 (^*^)	223.13
	MARC_P6-10	2.01 (^*^)	221.95		
	MARC_P+10	1.98 (^*^)	218.29		
	JCB_BLB	3.04 (^*^)	227.89		
	D2_D2BQ	2.58 (^*^)	226.94		
	D2_D2S	2.26 (^*^)	224.38		
**IV**	MIN_Q1-2	2.77 (^*^)	359.2	MIN_Q1-2	2.38 (^*^)	303.35
	MARC_G6-10	3.68 (^*^)	335.69		
	MARC_EMP	3.28 (^*^)	345.7		
	FIGURE			FIGURE		
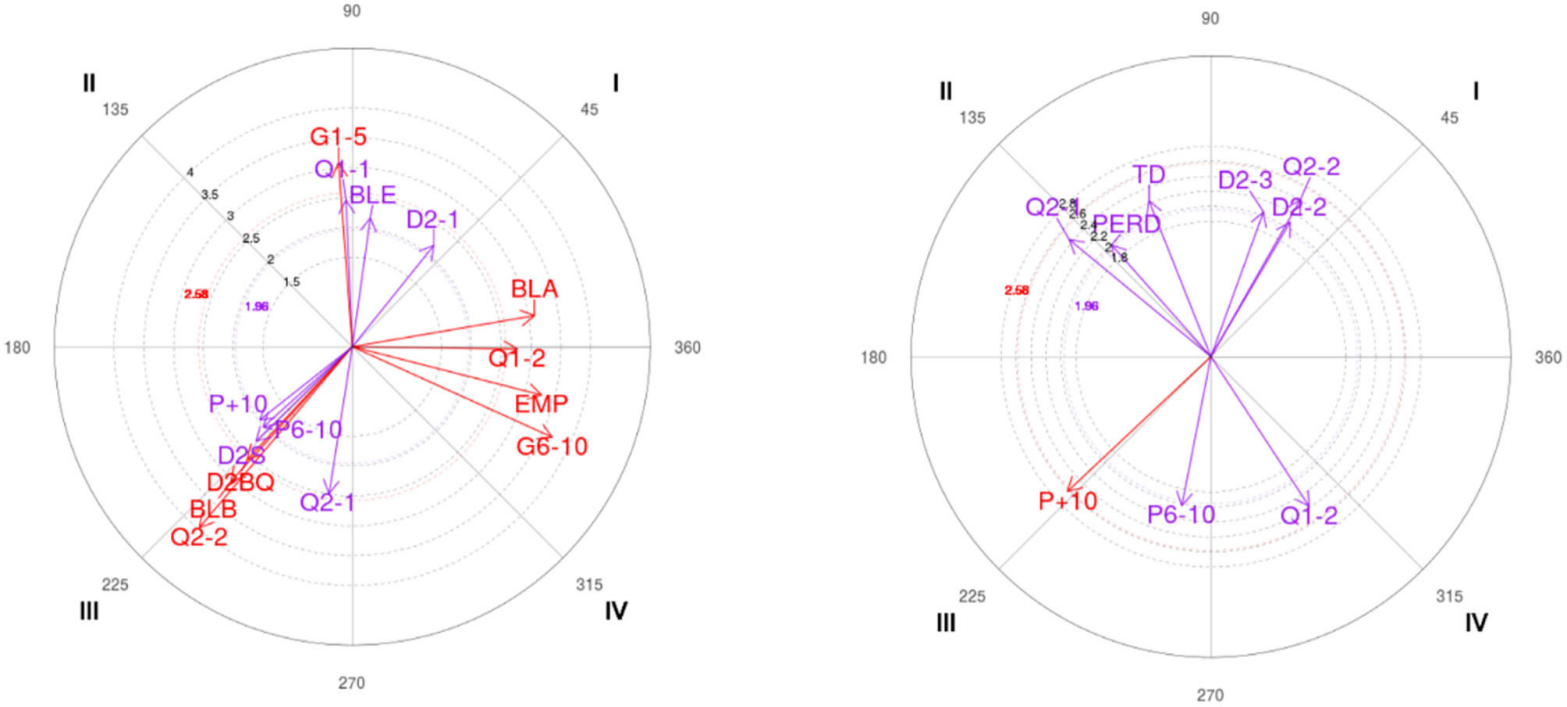						

*Significant relationships*:

* *p < 0.05 vector ≥1.96*.

Related to the starting focal behavior “on the opposite side of the ball screen” (SCBQ), the results show the following significant relationships in quadrant I: BLE (guard with the ball), BLA (forward with the ball), and D2-1 (defender close to the player with the ball) for the male category; and Q2-2 (from min 5 to 0 of the second quarter), D2-2 (defender behind the screener), and D2-3 (defender behind the three players) for the female category. In quadrant II, Q1-1 (from min 10 to 5 of the first quarter) and G1-5 (winning by between 1 and 5 points) are shown as significant behaviors for the male category; and for the female category Q2-1 (from min 10 to 5 of the first quarter), TD (disadvantageous shot) and PERD (loss of possession). In quadrant III, the pairing behaviors P6-10 (losing between 6 and 10 points) and P+10 (losing more than 10 points) are significant for both the male and female categories. In the male category, the behaviors Q2-1 (from min 10 to 5 of the second quarter), Q2-2 (from min 5 to 0 of the second quarter), BLB (base with ball), D2BQ (defender stays in the ball screen), and D2S (defender chases) are also significant. Finally, quadrant IV shows the conditioned behaviors G6-10 (winning from 6 to 10 points) and EMP (tie) for the male category, and Q1-2 (from min 5 to 0 of the first quarter) for both categories.

Table 6 below shows the results of the polar coordinate analysis for the SLBQ focal behavior (ball screen side exit).

**Table 6:** Significant relationships and vector representation between SLBQ focal behavior and mating behaviors for each gender.

**C**	**Male**			**Female**		
	**Category**	**Radio**	**Angle**	**Category**	**Radio**	**Angle**
**I**	MIN_Q2-1	3.13 (^*^)	68.97		
	MIN_Q2-2	3.79 (^*^)	37.63		
	JCB_BLB	2.87 (^*^)	65.34		
	D2_D2BQ	1.98 (^*^)	0.69		
	D2_D2S	2.45 (^*^)	42.27		
**II**	MARC_G6-10	3.1 (^*^)	139.82	MIN_Q4-1	2.32 (^*^)	137.86
	MARC_EMP	2.96 (^*^)	164.18		
	D1_D1C	2.29 (^*^)	159.1		
**III**	MIN_Q1-1	2.95 (^*^)	227.47	MIN_Q2-2	2.01 (^*^)	214.17
	JCB_BLA	2.36 (^*^)	192.91	MARC_G6-10	2.08 (^*^)	265.79
	D2_D2-1	2.72 (^*^)	201.99		
**IV**	MARC_G1-5	3.18 (^*^)	271.44	MIN_Q2-1	2.48 (^*^)	333.11
	JCB_BLE	2.57 (^*^)	272.55	MIN_Q3-2	2.01 (^*^)	331.47
				A1_A1E	2.03 (^*^)	336.21
				FIN_PERD	2.39 (^*^)	320.75
	FIGURE			FIGURE		
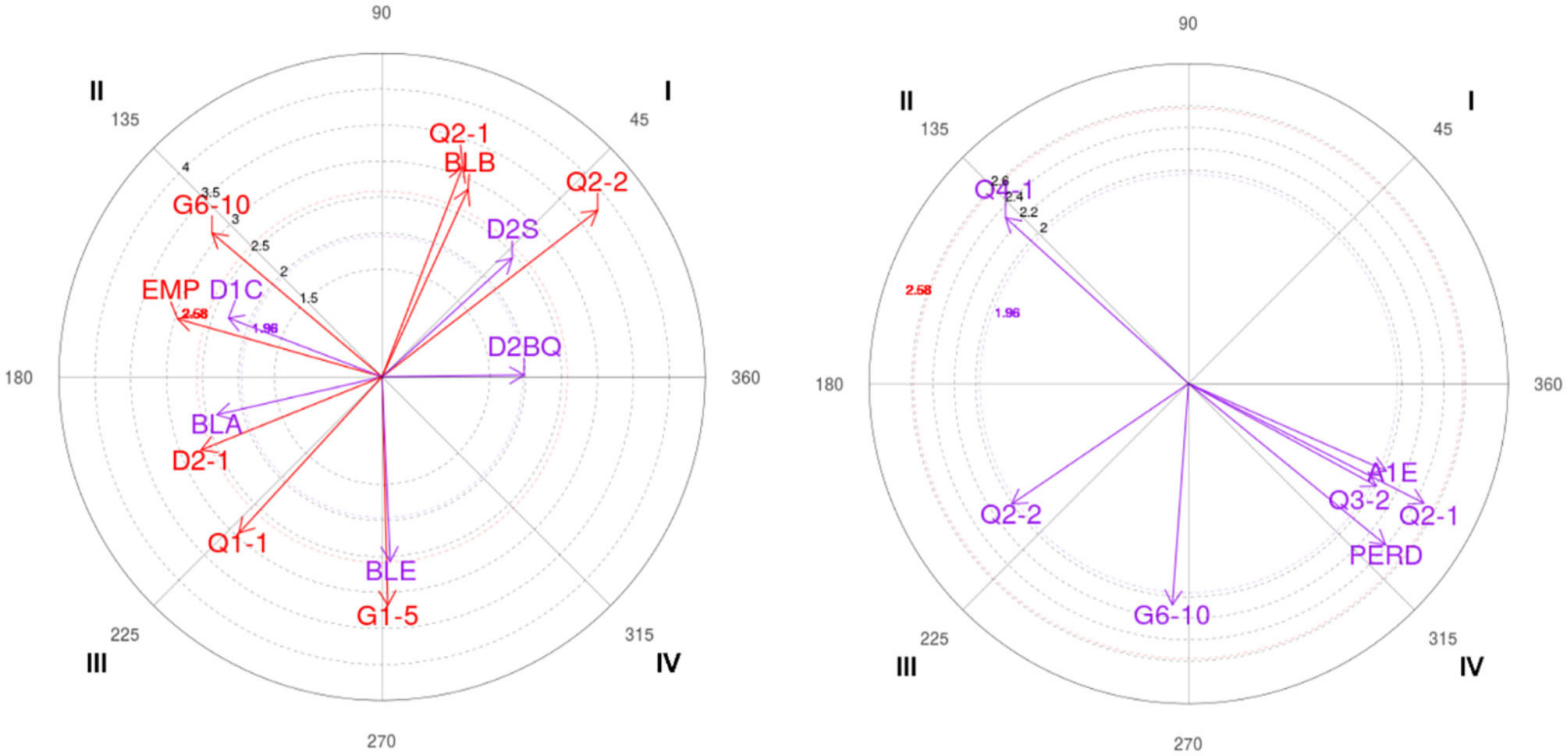						

*Significant relationships*:

* *p < 0.05 vector ≥1.96*.

The results of the polar coordinates analysis for the focal behavior of the SLBQ show several significant relationships for each category. In the first quadrant, for the male category, behaviors Q2-1, Q2-2, BLB, D2BQ, and D2S are significant. In contrast, the female category does not show significant relationships with any of the mating behaviors. In quadrant II, the significant conditioned behaviors are: G6-10, EMP (tie) and D1C (defender changes defender) for the male category; and Q4-1 (from min 10 to 5 of the fourth quarter) for the female category. In the third quadrant are Q1-1, BLA, and D2-1 (defender sticking to the player with the ball) for the male category, and Q2-2 and G6-10 for the female category. Finally, in quadrant IV, the behaviors G1-5 (winning between 1 and 5 points) and BLE (guard with the ball) are significant for the male category; and Q2-1 (from min 10 to 5 of the second quarter), Q3-2 (from min 5 to 0 of the third quarter), A1E (attacker makes a shot), and PERD (loss of possession) for the female category.

### Data Mining

An analysis technique known as clustering has been used, which allows the identification of typologies or groups where the elements are very similar to each other and very different from those of the other groups. K-means algorithm has been used for this clustering, which is an algorithm classified as a partitioning and repositioning method. This algorithm is so far the most widely used in scientific and industrial applications. The name comes from the fact that it represents each of the clusters by the average (or weighted average) of its points, i.e. by its centroid. The centroid representation has the advantage that it has an immediate graphical and statistical significance. The sum of the discrepancies between a point and its centroid, expressed through the appropriate distance, is used as the objective function. The objective function, sum of the squares of the errors between the points and their respective centroids, is equal to the total variance within the cluster itself. The sum of squares of the errors can be rationalized as the negative of the log-likelihood for mixed models using normal distributions.

Each cluster represents a “quotient set.” Table 7 shows cluster 0 as the most representative cluster.

**Table 7:** Results of the clustering of data.

**Attribute**	**Cluster#0**	**Cluster#1**	**Cluster#2**	**Cluster#3**
	**(125.0) (43%)**	**(60.0) (21%)**	**(61.0) (21%)**	**(45.0) (15%)**
MARC	G1_5	P1_5	P6_10	P6_10
TIP.BQ	SIMP	SIMP	SIMP	SIMP
JCB	BLB	BLB	BLB	BLB
JBQ1	BQ1P	BQ1P	BQ1P	BQ1P
JBQ2	NBQ2	NBQ2	NBQ2	NBQ2
ZBQ	Z2	Z3	Z3	Z2
SD	IND	IND	IND	IND
ORIBQ	OB	OC	OB	OB
LBQ	BQLND	BQLD	BQLND	BQLD
SBQ	SLBQ	SLBQ	SLBQ	SLBQ
D1	D1C	D1N	D1N	D1C
D2	D2_1	D2_2	D2_1	D2_1
A1	A1P	A1P	A1BM	A1BM
A2	A2PR	A2PP	A2PR	A2PR
FIN	CONT	CONT	CONT	BV
SEX	WOMAN	WOMAN	MAN	MAN

The results show that most of the players, regardless of gender, usually make simple ball screens, the player with the ball is the point guard, the screener is the center, there is no second ball screen, the ball screen zones are usually zones 2 and 3, the defensive system is individual, and the defender is close to the player with the ball.

In women's basketball, normally the attacker having the ball passes it and the second attacker makes a pick and roll or pick and pop. Looking at male players, the attacker having the ball hands it and the second attacker usually makes Pick and Roll. Normally, the situation is completed by continuation of the play in women; whereas men either continue the play or the point guard is the player who creates advantage.

**Figure 1:**
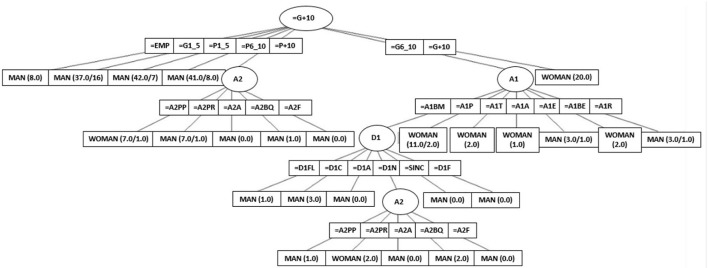
Decision tree.

The gender decision tree as a function of factor challenge attributes has a hit ratio of 80%, and it is shown in Figure 1.

The attributes that produce a significance in the sex of the player stand out; these are: MARC (the final score), A1 (ATTACKER 1), A2 (ATTACKER 2), and D1 (DEFENDER 1). Specifically, an interesting result is that only female teams win scoring 10 points more than the other team, but when wining scoring is more than 6 and less than 10 points than the other team, the behavior of attacker 1 is the factor determining the gender. Only female attacker 2 will make a pick and pop compared with a male attacker.

## Discussion

The objective of this work is to identify, by means of data mining and polar coordinates analysis, patterns in the recorded data to explain and predict the behaviors occurring in the execution of the tactical means of ball screen in professional basketball teams in Spain, differentiating it according to gender.

The *ad hoc* observation tool created has passed the data quality and generalizability analysis tests, which allows a reliable recording of the behaviors appearing during the game action. In addition, it has been shown that polar coordinates analysis is a useful technique for the study of the relationships established between behaviors in a tactical situation in competitive team sports (Vázquez-Diz et al., 2019), and also in data mining analysis to show useful information on how scores are distributed and groups are classified (Art Data Mining and Mixed Methods).

Thus, the results of the different polar coordinates analyses have shown statistically significant differences in mating behaviors between the male and female categories for each of the focal behaviors analyzed.

Ball screen is one of the most used collective offensive resources, present in ~30–45% of positional attacks (Nunes et al., 2016; Romarís Durán, 2016), reaching in male categories an efficiency percentage of 45% in attacks in which ball screen is used as a finishing action. In female category they obtain 46% efficiency, a result that is above the average of positional attacks; so something more should be taken into account in the completion of these attacks (Romarís Durán et al., 2013).

On the other hand, the presence of ball screens in the transition game in Liga Femenina is very scarce; they are used only in 8%. However, ball screen is presented in 31.2% of transitions to take advantage or causing more advantage in defensive disorganization, reaching an efficiency percentage of ~70% (Romarís Durán, 2016).

Depending on the analysis and interpretation of the data, differences between male and female categories are found in the mating behaviors that show significant relationships with the exit on the opposite side of the ball screen. Although in the male category, the exit on the opposite side of the ball screen shows a relationship of mutual excitement with the defender close to the player with the ball, in the female category the relationship occurs when the defender passes behind the screener and behind the three players. These results coincide with the work conducted by Battaglia et al. (2009) who determined that the most effective defenses performed by the defender of the screened player are to pass behind the player with the ball (pass from second) and to pass behind the three players involved in the ball screen (pass from fourth).

The opposite side of the ball presents a relationship of mutual excitement in the male category with the shooting guard as the attacker with the ball (BLE) and the small forward (BLA), who are players with a percentage of success in the three-point shot of 34.96% and 33.68%, respectively (Arjonilla López, 2010). This could explain why the defender of the player with the ball leaves the ball screen close to the player with the ball, since it makes it difficult for the attacker to shoot.

On one hand, both categories show a mutually inhibitory relationship between the output on the opposite side to the ball screen and the marker against (P6-10 and P+10), and on the other hand, in male category the marker in favor (G6-10) is inhibited (IV quadrant). A study has shown that the use of pick and roll with the unfavorable marker is bigger (Nunes et al., 2016), but it is unknown if that study took into account ball screens where the player with the ball did not come out on the side of the ball screen.

Looking at the results obtained with the focal behavior, output on the ball screen side and no significant relationships of mutual arousal were found in the female category. On the other hand, a mutually excitatory relationship is shown with the defender chasing and with the defender pinned on the ball screen side for the male category. In addition, in male category the focal behavior is linked inhibiting the marker in favor (G1-5), which could be because of the fact that the use of pick and roll is smaller for a favorable score (Nunes et al., 2016), and in female category it is linked inhibiting the completion of the play by a loss (PERD), which is consistent with the study conducted by Romarís Durán (2016) in which the results showed that ball screen obtains an efficiency of 46%.

The results of the data mining analysis allow to complement and deepen the knowledge of the relationships established between behaviors, showing specifically the tendency to start or finish for each gender in the development of the game situation studied. The female category shows more tactical variety on the part of the second attacker by performing pick and roll or pick and pop at the beginning of the action, whereas the male category focuses on the pick and roll. In the completion of the play, the role of the point guard stands out in the male category as the player who creates advantageous situations for the team in this specific tactical situation.

The study has shown all the virtues of the analysis techniques used; however, it also shows limitations that should be considered, such as the lack of similar studies that would allow the information obtained to be contrasted and compared. Another limitation is not being able to extrapolate the results to competitions in other countries because all the analyzed games belonged to the Spanish basketball leagues (ACB and Liga Femenina).

The findings of both analyses suggest that these techniques are suitable to be applied in sport psychology, specifically to explore different variables that are usually studied in this field of knowledge.

## Data Availability Statement

The raw data supporting the conclusions of this article will be made available by the authors, without undue reservation.

## Author Contributions

AH-M, VM-S, RR, JP-B, JM-B, CF, BT-G, and JV-D participated in the study design and data collection, performed statistical analyses and contributed to the interpretation of the results, wrote the manuscript, approved the final manuscript as submitted, and reviewed and provided feedback to the manuscript. All authors made substantial contributions to the final manuscript.

## Conflict of Interest

The authors declare that the research was conducted in the absence of any commercial or financial relationships that could be construed as a potential conflict of interest.

## Publisher's Note

All claims expressed in this article are solely those of the authors and do not necessarily represent those of their affiliated organizations, or those of the publisher, the editors and the reviewers. Any product that may be evaluated in this article, or claim that may be made by its manufacturer, is not guaranteed or endorsed by the publisher.
